# A Plea for the Earlier Diagnosis of Appendicitis1Read at the thirty-ninth annual meeting of the Medical Association of Central New York, at Syracuse, October 16, 1906.

**Published:** 1907-02

**Authors:** Albert L. Beahan

**Affiliations:** Canandaigua, N. Y.


### A Plea for the Earlier Diagnosis of Appendicitis.[1]

By ALBERT L. BEAHAN, M. D., Canandaigua, N. Y.

THERE is no abdominal disease of frequent occurrence that is of such virulent type as appendicitis. It comes usually during the night and strikes its victim with a sudden attack and with relentless cruelty. The storm spoils the appendix, leaving it a blackened, disorganised object of putrid material or some lesser pathological character, which nature may succeed in covering with bowel, or omentum, or cause it to adhere to the abdominal wall and shut off the infective area. There is need to have a line on the diagnosis of appendicitis and to be sure to know where to let that line fall.

Some of the cardinal points in the diagnosis of appendicitis are established. Sudden pain over the epigastrium, which gravitates to the right iliac region; nausea of a peculiar type that is easy and of startling quickness, to be succeeded by vomiting, being like the vomiting of pregnancy and thus showing its reflex character, are always known signs of appendiceal inflammation. To these add the rigid right rectus muscle with right side tenderness at or near McBurney's point, and the classical picture of appendicitis is produced.

What other guides to a correct diagnosis do we have? A halting gait, the right leg being dragged or stiffened when locomotion is attempted, is a very helpful aid, and should always be studied in obscure ambulatory cases. It is one of the old Potts abscess signs and was probably an unappreciated evidence of abscess about the appendix, as the old cold abscess of the right groin has been found in nearly 100% of cases to be of appendiceal and not of tubercular origin. The chronic sepsis of a pus inclusion in appendicitis gives a dirty, sallow complexion. Intestinal indigestion with gas formation, frequent colic, inability to partake of uncooked food wthout abdominal distention, is significant of carrying a diseased appendix.

---

1. Read at the thirty-ninth annual meeting of the Medical Association of Central New York, at Syracuse, October 16, 1906.

To these considerations the mind turns when the problem of diagnosis is to be solved. The relation of success to operative intervention demands the early diagnosis of this disease. The plea for its necessity is not born of any despair from recent experience. The contrary is true, as nearly five years of our own hospital life have passed with a mortality of but one case, which occurred on August 25, last, and that was a case of black easy vomiting, with pus from the ensiform to the pubes when the patient was admitted to the hospital.

The reason for the emphasis of early surgical measures is obvious to the experienced observer. To the physician who follows his cases to the operating table and there corrects his opinion, a fairly reasonable hope may be felt that observation can be depended upon to know when danger from appendicitis is imminent. But the power of analysis by the mind is not always keen. Experience must teach hard lessons to each practitioner of medicine. Only a few weeks since a case of appendicitis was mistaken for cholera morbus, and the patient insisted on walking some distance even while the pelvis contained the products of a ruptured appendiceal abscess, and three pitchers of sterile water were required to make a clean abdominal toilet. We apologise for so late a diagnosis.

Another recent case points a moral. The patient experienced a number of attacks of appendicitis during the past seven years. Last April a sharp disturbance occurred. When asked what his attending physician did for him, he replied that he emptied his stomach and gave him a hypodermic of morphine. The pain of an attack of appendiceal inflammation of simple type, can be relieved by calomel, castor oil and salines, even very speedily. If these measures do not relieve, the dictum should be to operate at once. It is criminal practice to benumb the cerebrum with morphine or other opiates for belly pains. No individual experience of itself can guide safely.

The outcome of operations by the abdominal route demands an early diagnosis of appendicitis. Before rupture of the appendix every case should recover within twenty-one days. After rupture every hour of exposure of the intestinal surfaces with their lymphatics, to the consequences of septic infection, lessens with an increasing degree the chance of recovery. The line of incision is usually infected in delivering the appendix and union is by slow granulating process. More or less tardy convalescence is to be anticipated. Another heresy that demands puncturing is, that the pulse must be relatively rapid and the temperature high to warrant a statement that the early diagnosis and early operative interference are important. If the appendix points among the small intestines, or bends up over the ileocecal valve where the lymphatic glands are numerous, the temperature, indicating rapid inoculation of septic toxins, will be high and the pulse will respond as an inflammatory index. But if the appendix is against the abdominal wall, as say from an old attack with adhesions, or if it lays on the shelf formed by the iliac bone, just above Poupart's ligament, simulating and puzzling one to determine if it might not be enlarged inguinal glands, in these instances an ugly mixed infection may develop with but little constitutional disturbance.

Only a small percentage of appendicular inflammations can be diagnosticated in the early stages by pulse and temperature indications. If the appendix points away from lymphatic gland regions an abscess must form, as in a gangrenous inclusion, and rupture occur through the first line of defense, the first barrier wall, and septic products will leak into the abdominal cavity and reach the lymph channels before inflammatory signs show in pulse and temperature. It is safer not to trust the count of the pulse and not to put the thermometer into commission in settling an early diagnosis of appendicitis. To appreciate what serious changes occur in a few hours when symptoms are not over evident, one must often look into the open abdomen and study the pathology and correct the judgment by visual examinations.

The thickened infiltrated cecum, the separation of muscular and serous coats by a dissecting gangrenous process, the patches of lymph membrane, of diphtheric appearance, the binding together of bowels, the mothering omentum caring for a pocket of filth with sacred fidelity in simple cases, the petechial blood points on the peritoneal surface betokening a hemorrhagic infarction, or an ulcerated patch underneath in the mucous membrane of the bowel; these various phenomena give a true picture of the case when contemplated through the open incision. There is no other way to study appendicitis. Theorising is useless. Temporising with cold storage or salad oil, is foolhardy, even criminal. Pathological changes go on, even if ice is a few inches above the diseased section and the cecum can be unloaded to relieve the gas pressure, but acute and, later, chronic stricture prevents the normal appendiceal current from discharging its contents very completely.

In some subjects pain is not great, depending on the nervous development of the individual, but pain must remain the chief guide. Unrelieved tenderness with exacerbations of pain are of some importance, but sudden cessation of pain signifies the gangrenous condition. During the month of August, last past, five cases of appendicitis entered the Canandaigua Hospital, having ruptured appendiceal abscesses of four to twenty-four hours'

duration, with pus in the pelvis and intestinal folds. They were all of the desperate sort and occurred in a period of weather when great humidity and high temperature were giving general bowel troubles of deep mucous membrane type, dysentery prevailing, and great prostration of the sick was noticeable. Each of these cases had a gangrenous appendix with abscess. I dawdled with one of the cases for need of an early diagnosis. He walked with no hitch of right leg, which fact was explained when we found free pus, and appreciated that both recti muscles and both psoas muscles were splinting the inflamed intestines, and we recalled that he stooped when walking and really limped, or dragged both legs in a peglegged sort of gait. He was a foreigner and this obscured the history and salved professional pride.

The early diagnosis of appendicitis is not made with the hypodermic syringe and morphine, not with calomel and salts, not with ice packs, starvation and rectal feeding, not with salad oil or castor oil. It is made by watching at a distance from the patient the tense face of distress. By the history of gastric disturbance, settling toward the navel and right iliac region; by easy vomiting. It is settled by the finger tips, appreciating that the right or left rectus, or both, are harder than normal, and by finding a sore spot at the right side of abdomen, somewhere below the liver, above Poupart's ligament, and without the umbilicus. It may be added that rest and procrastination are not available aids. In the last five years, or nearly so, no appendicitis case remains in our hospital to exceed an hour, before the appendix is removed. Probably more night than day operations are done. The month prior to establishing this rule we had a mortality of two and we believe one of these was due to waiting over night before operating. To the physicians who are so alert and bring their patients to the hospital so promptly, is due much of the credit of a normal or no mortality result.

With such flattering results, associated with a definite plan, errors must lessen. A saving technic is required to win desperate cases and establish in the minds of the profession and the laity, a confidence in operative interference. Then the appreciation that better results can be had if the appendix were removed earlier follows as a natural reasoning, and the community is won for the scientific surgical position.

18 Gorham Street.

---

Eczema of the breast should always be viewed with suspicion, for it may be a symptom of Paget's disease and percursory to cancer. In these cases the growth may for a long time appear as a superficial ulcer, and thus lead to errors in diagnosis.—*International Journal of Surgery.*

